# Changes in Fracture Epidemiology Due to COVID-19 Crisis; a Letter to Editor

**Published:** 2020-04-21

**Authors:** Seyyed Saeed Khabiri, Mohammad Hossein Nabian, Heydar Zeynolabedin, Javad Veisi, Vahid Rastgou, Mehdi Naderi, Shokofeh Maleki

**Affiliations:** 1 Department of Orthopedic Surgery, Clinical Research Development Centre, Taleghani and Imam Ali Hospitals, Kermanshah University of Medical Sciences, Kermanshah, Iran.; 2 Department of Orthopedic Surgery, Joint Reconstruction Research Center, Tehran University of Medical Sciences, Tehran, Iran.; 3 Department of Orthopedic Surgery, Faculty of Medicine, Kermanshah University of Medical Science, Kermanshah, Iran.; 4 Clinical Research Development Centre, Taleghani and Imam Ali Hospitals, Kermanshah University of Medical Sciences, Kermanshah, Iran.

**Dear Editor**

Since December 2019, when the first case of COVID-19 was reported in China, the main strategy of health policy makers has been to quarantine and impose social restrictions, causing significant behavioral changes in people due to fear of infection (1, 2). Laws limiting traffic, reduced travel permits, paying attention to personal hygiene, and making efforts to clean up private and public environments are some of the changes that have been observed. We also noticed alterations in patients' admission to trauma centers.

In a retrospective cross-sectional study, demographic characteristics and type of fracture were analyzed in patients referring to the trauma center of Taleghani Hospital, Kermanshah, Iran, from 1^st^ March to 15^th^ April, in 3 consecutive years (2018 to 2020).

**Figure 1:**
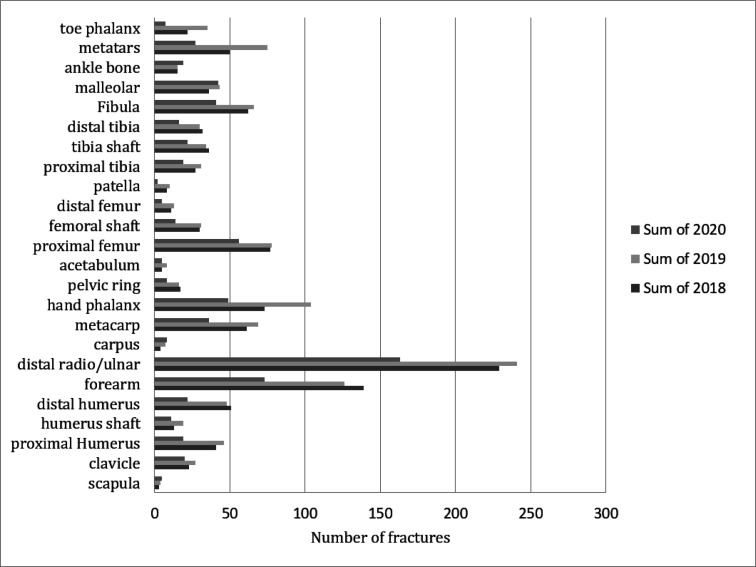
Types and number of fractures during the COVID-19 outbreak (March and April of 2020) and similar periods in 2018 and 2019

2,483 trauma patients with the mean age of 37.31 ± 22.86 years were studied (Table1). The findings showed that, the number of fractures has generally decreased in March and April 2020. The change is more prominent in children and young men aged 18 to 35 years, which may be due to reasons such as schools being closed, sports activities being ceased, and the decrease in traffic, as well as the reduction of accidents that have affected these age groups. The age, gender, and type of fracture had approximately the same pattern during the studied period in three years, but we have seen an increase in some specific fractures such as foot bone and ankle (figure1). The fractures of middle-aged women, such as those with osteoporosis, like proximal femur, humerus, and distal radius, seem to have decreased in number during this period.

In the study of scott et al., during quarantine and social distancing, despite the decrease in the total number of patients, fragility fracture statistics had remained the same (3). Also, in the study of chui et al., they noted that due to cancellation of elective surgeries and the reduction of hospital workload, patients with pelvic fractures will be able to receive preoperative care faster. And better care has been provided for these patients during the COVID-19 era (4).

 Indoor layout optimization and strengthening the muscles to prevent falling and care for high-risk people is important. A program to treat osteoporosis should also be actively implemented in people over the age of 65.

It could be concluded that during the Corona era, because of the behavioral change of the people and the decrease in traffic, we have witnessed a decline in hospital referrals due to trauma and also a decrease in the incidence of fractures, especially those related to accidents. This report may help guide the efforts to improve the healthcare system for crisis preparedness and assist in allocating resources for treatment and predicting workloads.

**Table 1:** Number of cases referred to the emergency department with fractures based on gender and age in the studied years

**Characteristic**	**March 1** ^st^ ** to April 15** ^th^		
	**2018**	**2019**	**2020**
**Fracture**			
Number	957	948	578
**Gender**			
Male	605 (63.2)	602 (63.5)	393 (67.9)
Female	352 (36.8)	346 (36.5)	185 (32.1)
**Age**			
Mean ± SD	35.41±22.65	37.97±23.15	39.37±22.51

SD: standard deviation.
